# Socio-demographic drivers of household food waste management practices in Thailand

**DOI:** 10.1371/journal.pone.0321054

**Published:** 2025-04-01

**Authors:** Patranit Srijuntrapun, Pattama Ket-um, Witsanu Attavanich

**Affiliations:** 1 Department of Education, Faculty of Social Sciences and Humanities, Mahidol University, Nakhon Pathom, Thailand; 2 Srinakharinwirot University, Nakhon Pathom, Thailand; 3 Department of Economics, Faculty of Economics, Kasetsart University, Bangkok, Thailand; Bina Nusantara University, INDONESIA

## Abstract

**Objective:**

The escalating food waste crisis, with millions of tons of food being discarded annually, highlights the pressing necessity to improve household food waste management practices. This complex and multifaceted challenge is a crucial element of a comprehensive national strategy for reducing food waste. This article seeks to examine the diverse demographic and social factors that shape household food waste management practices in Thailand.

**Methods:**

A substantial national dataset (n =  2,500) was meticulously gathered through questionnaires, using multi-stage sampling and multiple regression analysis to reveal critical insights.

**Results:**

This study reveals that educational attainment (β =  0.299), household size (β =  0.201), and monthly income (β =  0.058) are positively associated with effective household food waste management practices. Notably, the type of housing, such as single houses over 200 square meters (β = .058**) and condominiums/apartments (β = .063**), significantly influence food waste management behaviors. However, townhouses (β =  -.074***) are negatively associated with improved food waste management practices. The research also identifies key barriers to effective food waste prevention, including the lack of organizational guidance (29.4%), the perception that waste reduction does not save costs (26.1%), and uncertainty about where to donate surplus food (25.2%). Additional challenges of managing food scraps include the uncertainty about options for donation or sale of food scraps (43.3%) and the limited knowledge of composting or bio-fermentation methods (30.2%).

**Conclusions:**

In conclusion, this study provides essential insights for policymakers, practitioners, and researchers by identifying key demographic, knowledge-based, and behavioral factors that shape household food waste management. The study’s findings underscore the need for targeted educational initiatives and infrastructure enhancements. Policymakers can leverage these insights to develop policies that support public-private partnerships and improve waste management infrastructure. Practitioners can apply this knowledge to implement more effective waste segregation strategies, while researchers are encouraged to explore socio-economic factors influencing food waste at a national scale, thereby addressing critical research gaps. This comprehensive approach is vital for reducing household food waste and promoting sustainable waste management practices across diverse communities.

## Introduction

Reducing household food waste presents a critical challenge for sustainable development and the growth of the circular economy. This strategy emphasizes the continuous use of resources, minimizing waste through prevention, and using surplus food for productive purposes like animal feed, bioprocessing, and efficient recycling [1]. It is aligned with Sustainable Development Goal (SDG) 12.3, which targets a 50% reduction in global food waste per capita at both the retail and consumer levels, while simultaneously reducing food losses across the supply chain by 2030 [2].

Recent data highlights a concerning surge in household food waste, which now constitutes nearly one-third of all household waste. According to the FAO [2], about 30% of the global food supply is lost or wasted each year. Globally, households contribute approximately 931 million metric tons of food waste, accounting for 17% of available food. China and India are the largest contributors, with 92 million and 69 million metric tons of food waste respectively. Interestingly, food waste per capita remains similar between developed and developing countries, with West Asia and Sub-Saharan Africa showing the highest per capita rates [3]. This waste has profound environmental implications, as decomposing food in landfills releases methane, a potent greenhouse gas that significantly contributes to climate change [4,5,6,7]. Beyond its environmental costs, water, energy, and labor spent to produce wasted food are also lost, depleting valuable resources and worsening environmental degradation [8,9]. Addressing household food waste is thus critical, not only for reducing waste but also for mitigating its broader environmental impacts.

Tackling this issue requires a multifaceted approach that integrates education, policy, and community engagement. The food waste management hierarchy, developed in recent years, has emerged as a global guideline, prioritizing the prevention and reduction of waste at the source and emphasizing reusing, recycling, treating, and ultimately disposing of waste, leaving landfilling as a last resort. Within this framework, food waste management practices encompass individual and household behaviors, routines, and systems, such as effective meal planning, food waste recycling, and the separation of organic waste for composting. All these practices aim to reduce food waste as much as possible, with the goal of achieving zero waste levels. Developing effective interventions requires a deeper understanding of household behavior and the socio-demographic factors that influence food waste management. Research shows that factors such as household size [10], income [11], and education [12,13] are crucial determinants of food waste behavior. For example, single-person households tend to waste more food due to limited economies of scale and less efficient food management routines [14]. Other key drivers include food waste knowledge, moral standards, eating habits, and routines for reusing leftovers [15]. Additionally, socio-economic status and the type of housing play a significant role in determining both the volume of waste generated and the methods employed for its management [16].

In Thailand, the challenge of food waste is particularly pressing. A 2022 study by the Pollution Control Department identified food waste as the most prevalent form of waste in disposal sites [17]. According to UNEP’s 2024 Food Waste Index Report, the average Thai citizen produces 86 kilograms of food waste per year [1]. However, much of the existing research on food waste in Thailand focuses on urban areas or specific population segments, leaving a gap in the national understanding of food waste behavior. This gap underscores the need for a comprehensive nationwide study to better understand household food waste behavior and the socio-economic factors driving it, particularly in the context of Thailand’s unique culture and economy.

This study examines the socio-economic factors influencing household food waste behavior in Thailand by analyzing survey data collected from 2,500 respondents via multistage cluster sampling to ensure representativeness. The questionnaire addressed the socio-economic characteristics, knowledge, and food waste management practices of the participants. This data was analyzed using multivariate linear regression to identify key behavioral predictors. The findings of this study should contribute to advance knowledge in this area. This study also seeks to deepen the understanding of emotional and perceptual factors influencing consumer participation in food waste reduction, as a foundation for targeted interventions and policy recommendations. Importantly, the data obtained from respondents across the country have led to the formulation of Thailand’s first food management plan, namely the Food Waste Management Plan (2023–2030) and the Food Waste Management Action Plan Phase 1 (2023–2027) [17]. The study’s novelty lies in its use of the internationally recognized food waste hierarchy framework and a comprehensive national dataset to analyze socio-demographic characteristics and knowledge factors influencing food waste behavior in Thailand. It identifies specific groups in need of targeted policy interventions, contributing to the development of Thailand’s first comprehensive food waste management framework. The findings offer valuable insights for policymakers and practitioners to foster sustainable behaviors, advance SDG 12.3, and promote a circular economy.

## Methodology

### Study area

In 2023, Thailand produced 26.95 million tons of municipal solid waste, averaging 73,840 tons per day, a 5% increase from the previous year. The average waste generation rate stood at 1.07 kilograms per person per day. Out of this, 15.64 million tons were processed in 2,079 waste treatment facilities. However, only 114 of these facilities operated in accordance with scientifically recognized waste management principles. These included 73 sanitary or semi-aerobic landfills, 7 waste-to-energy incinerators, 3 incinerators with pollution control, 3 composting or biogas systems, 5 refuse-derived fuel (RDF) production systems, and 23 integrated systems. The remaining 1,965 sites operated as open dumps or controlled landfills, including 77 incinerators lacking pollution control. Major issues persist in relation to inefficient waste separation at the source, exacerbated by the low resale value of certain packaging materials and the absence of enforced legislation mandating waste separation or imposing penalties for non-compliance. As a result, local governments are burdened with high waste management costs, limiting the funds available for proper waste disposal and causing many facilities to function improperly [17].

The Pollution Control Department has identified food waste as the most prevalent form of waste in Thailand, making up 38% of all municipal solid waste—an average of 9.68 million tons, 39.5% of which remained edible [17]. The 2024 UNEP Food Waste Index Report estimated that Thailand generated 86 kilograms of food waste per person annually [1]. In 2023 alone, Thailand produced 10.24 million tons of food waste, with an average of 155 kilograms per person per year. This waste comprised 40% edible food and 60% inedible parts, such as bones and shells. Food waste primarily resulted from improper trimming, cooking, and storage, which led to spoilage before use. Efforts have been made to address this issue through local government initiatives, such as the “Food Waste Bin for Global Warming Reduction” project, which promotes greater separation and utilization of organic waste and food scraps. These programs have shown some progress, with improved waste separation and utilization rates compared to previous years [17].

### Data collection and characteristics of participants

This study used a quantitative approach to gather data from households across Thailand between 1 March and 31 July 2021. The data was originally collected by the Pollution Control Department and GIZ, which were commissioned to develop a national baseline roadmap. Drawing on demographic data from 21,884,396 Thai households [18], the sample size was calculated using Yamane’s formula [19], resulting in a sample of 2,500 households. This sample size was selected to provide a 98% confidence level with a 2% margin of error.

The study employed a multistage cluster sampling method with three key stages. In the first stage, Thailand was divided into five regions: Bangkok and its metropolitan area, the Central region (including the West and East), the North, the South, and the Northeast. In the second stage, two provinces were randomly selected from each of these regions. In the final stage, two areas were chosen from each province—one inside and one outside a municipal zone—using simple random sampling. The sample distribution by region is shown in Table 1.

**Table 1:** Sample size of the study by region (total: 2,500 questionnaires).

Region	Number of questionnaires	
	Online	Paper
Bangkok and its vicinity	300	33
Center (including West and East)	690	77
North	397	44
South	288	32
Northeast	575	64
**Total**	**2,250**	**250**

*Source: Authors*

Data collection was conducted using two methods. First, 2,250 households completed an electronic questionnaire (E-questionnaire). Second, face-to-face interviews were conducted with 250 households, representing 10% of the total sample. These face-to-face interviews, specifically targeted at participants living in zones with limited Internet access, were designed to ensure comprehensive coverage and representativeness of households in the study areas.

### Instruments

Based on the principles of the food waste hierarchy [20], the study’s questionnaire was carefully developed to collect comprehensive data on food waste management in Thailand. It was organized into four key sections:

1) General information: this section gathered the demographic details of respondents, including gender, education level, household size, household type, household income, and the identifiable causes of food waste.
2) Knowledge of food waste: participants’ understanding of food waste issues was assessed using dichotomous yes/no questions.
3) Food waste management behavior: this section evaluated food waste management practices using a Likert-type scale ranging from 1 (“Never”) to 4 (“Always”). The categories were defined as follows: “always” for habitual practice, “sometimes” for intermittent practice, “rarely” for occasional practice, and “never” for inexistent practice.
4) Barriers to food waste management: the final section explored the challenges that households faced in effectively managing food waste.

The dependent variable in the study was food waste management behavior, measured in terms of self-reported practices. Independent variables included socio-demographic factors such as gender, academic qualifications, household size, income, household size, and knowledge of food waste. These variables were selected based on prior studies which have demonstrated their influence on waste management practices. The questionnaire underwent a two-phase validation process. First, the questionnaire was developed and reviewed by five experts, who assessed and refined its content to ensure accuracy and relevance. These experts confirmed the validity of the questionnaire before it was deployed in the field. Second, a reliability test using a sample of 30 questionnaires was conducted to evaluate the instrument’s consistency and identify potential areas for improvement. Reliability was assessed at a 95% confidence level using Cronbach’s alpha, a widely accepted measure of internal consistency. Alpha values between 0.7 and 0.8 are generally considered acceptable for reliability [21]. The results of this assessment, which yielded an alpha coefficient, confirmed that the questionnaire was reliable and suitable for this study.

### Statistical analysis

The statistical analysis was conducted using SPSS (Statistical Package for the Social Sciences) version 20.0 (IBM Corporation). This analysis involved a descriptive examination of the respondents’ demographic characteristics, presented through frequency distributions and percentages. Key statistical measures such as the mean and standard deviation were also calculated. Furthermore, the relationship between socio-demographic variables and food waste management behavior was evaluated using correlation and multivariate regression analysis. A p-value of less than 0.05 was considered statistically significant, highlighting the importance of the findings.

### Ethical statement

This study received approval from Mahidol University Ethics Committee (reference number 2021/025.2302) before data collection was initiated. Participants were provided with detailed information about the study’s objectives and their rights to participate or opt out from it. Written informed consent was obtained from those households who chose to participate, confirming their agreement to complete the questionnaire. Participants were assured of the confidentiality of their responses, with a clear commitment that all collected data would be used solely for the purposes of this research.

## Results

### Demographic characteristics

This study is based on a comprehensive analysis of data collected from 2,500 respondents, representing households across the nation. The sample consisted of 1,750 females (70.0%) and 625 males (25.0%), while the remaining 125 respondents (5.0%) identified their gender as other. The majority of respondents, a total of 1,125 individuals (45.0%), held a bachelor’s degree, while 975 respondents (39.0%) had attained education beyond the bachelor’s level. In terms of household income, the largest group of 588 respondents (23.5%) consisted of those with a monthly income ranging between 15,001 and 30,000 baht ($424–$848). A notable segment of 492 respondents (19.70%) reported a monthly household income exceeding 75,000 baht (more than $2,121) (Table 2).

**Table 2:** Demographic characteristics (n = 2,500).

Demographic characteristics	Number	%
**Gender**		
Male	625	25.00
Female	1,750	70.00
Other	125	5.00
**Academic qualifications**		
No education (reference group)	25	1.00
Primary education	150	6.00
Lower secondary education	50	2.00
Upper secondary education	100	4.00
Diploma or vocational certificate	75	3.00
Bachelor’s degree	1,125	45.00
Postgraduate degree	975	39.00
**Marital status**		
Single	1,085	43.40
Married	1,242	49.69
Widowed or divorced	173	6.91
**Household size**		
1	239	9.56
2-5	1,925	77.00
6-9	311	12.44
10 or more	25	1.00
**Monthly income**		
Less than 15,000 baht ( <$424)	480	19.20
15,001–30,000 baht ($424-$848)	588	23.50
30,001–45,000 baht ($849-$1,272)	410	16.40
45,001–60,000 baht ($1,273-$1,697)	337	13.50
60,001–75,000 baht ($1,698-$2,121)	193	7.70
More than 75,000 baht ( > $2,121)	492	19.70
**Accommodation**		
Single house ( < 200 sq. m)	475	19.00
Single house ( > 200 sq. m)	1,375	55.00
Condominium/Apartment/Dormitory/Flat	225	9.00
Townhouse	200	8.00
Commercial building	100	4.00
Other	125	5.00

### Knowledge of household food waste management

The survey employed a series of yes/no questions specifically designed to evaluate the participants’ knowledge and understanding of household food waste management, with a focus on definitions, causes, and potential impacts. The findings indicate that respondents possessed a moderate level of overall knowledge (n = 1,493, 59.7%). A detailed analysis of individual questions reveals that a substantial majority of respondents correctly understood practices such as food preservation and processing (92.4%), sharing food with those in need (92.6%), composting food waste (97.6%), and using food waste for animal husbandry (97.1%). However, a significant proportion of respondents, as high as 71.9%, exhibited misunderstandings regarding the health risks associated with consuming food past the ‘Best Before’ date (Table 3). The reliability of these responses was deemed acceptable (α = .627).

**Table 3:** Knowledge of household food waste management (n = 2,500).

Knowledge question	True (*n*, %)	False (*n*, %)
Food waste consists of food scraps that are no longer edible.	1,055 (42.2)	1,445 (57.8)
Food that is still edible but is thrown away is not considered food waste.	1,670 (66.8)	830 (33.2)
Consuming food past the ‘Best before’ date can be harmful to health.	702 (28.1)	1,798 (71.9)
Food preservation and processing can reduce the amount of food waste generated in households.	2,310 (92.4)	190 (7.6)
Food waste has no impact on waste storage because it can be easily decomposed.	1,025 (41.0)	1,475 (59.0)
Wastewater from decomposing food discarded by households causes soil and water pollution.	2,127 (85.1)	373 (14.9)
Planning purchases and avoiding hoarding can help reduce food waste in households.	1,075 (43.0)	1,425 (57.0)
Sharing food with those in need can decrease household food waste.	2,315 (92.6)	185 (7.4)
Composting discarded food can further reduce household food waste.	2,440 (97.6)	60 (2.4)
Using discarded food to raise animals can reduce the amount of food waste in households.	2,427 (97.1)	73 (2.9)
Cronbach alpha = .627		

The majority of participants (n = 1,493, 59.7%) demonstrated a moderate level of knowledge, with scores ranging from 4 to 7. In contrast, 39.3% of them (n = 983) exhibited a high level of knowledge, scoring between 8 and 10. Only a small minority (n = 24, 1%) displayed low levels of knowledge, with scores ranging from 0 to 3.

### Household food waste management practices

Food waste management practices can be effectively categorized into two key areas: prevention and disposal. Prevention strategies involve a range of proactive measures designed to minimize waste, including planned purchasing, buying only what is needed, proper food storage, preparing suitable quantities, serving appropriate portions, and sharing surplus food. These actions not only help households to reduce waste but also to make savings on food costs. Conversely, disposal practices address unavoidable waste through management methods such as feeding excess food or scraps to animals, converting food scraps into compost or bio-fermented liquids, generating biogas from leftovers, or ensuring proper disposal.

Considering waste management behavior within the framework of the waste hierarchy concept, the study revealed that respondents made a moderate use of key prevention measures, including planned purchasing, purchasing food as needed, proper food storage, and serving portions as desired while sharing excess food. Certain preventive behaviors were commonly practiced, with participants all the time (48.1%) or often (33.2%) using existing ingredients to cook before acquiring new ones. Additionally, participants all the time (53.4%) or often (31.4%) prepared the right amount of food for their households.

Conversely, the study found a low incidence of behaviors related to the use and conversion of food waste. For instance, only 31.9% of respondents occasionally fed excess food or scraps to animals. Practices such as producing compost from food scraps were rarely undertaken, with 38.6% of participants never engaging in this activity. Similarly, 60.2% of respondents never processed food scraps into bio-fermented water, a potential natural fertilizer. In addition, most participants (77.9%) had never fed food scraps to earthworms to make nutrient-rich compost. Finally, a significant proportion of respondents (88.9%) indicated that they had never collected leftovers to produce biogas, a renewable energy source (Table 4). The reliability of these responses was acceptable (α = .853).

**Table 4:** Summary of household food waste management practices by frequency (n = 2500).

Practices	Always	Often	Sometimes	Never	($\bar{x}$)	Interpretation
**Planned purchasing**					**2.85**	**Moderate**
Regularly check the refrigerator and dry food locker before dispensing.	42.1 (1,052)	25.3 (633)	24.7 (618)	7.9 (197)	3.02	High
Write a food list of necessary food purchases before going to the market.	30.8 (770)	22.1 (552)	32.9 (823)	14.2 (355)	2.69	Moderate
**Purchasing food as needed**					**2.97**	**Moderate**
Buy food according to the planned purchase list.	32.1 (802)	33.6 (840)	25.1 (628)	9.2 (230)	2.88	Moderate
Purchase the right amount of food to avoid hoarding large quantities.	34.8 (870)	32.6 (815)	26.2 (655)	6.4 (160)	2.96	Moderate
**Proper food storage**					**2.98**	**Moderate**
Always store newly purchased food inside the refrigerator and move pre-stored food outside.	26.7 (667)	23.7 (593)	31.4 (785)	18.2 (455)	2.59	Moderate
Regularly check the expiration dates and prioritize using food before it expires.	59.2 (1,480)	23.1 (577)	12.9 (323)	4.8 (120)	3.37	High
**Preparing appropriate quantities of food**					**3.30**	**High**
Cook with existing ingredients before buying new ones.	48.1 (1,202)	33.2 (830)	14.6 (365)	4.1 (103)	3.26	High
Prepare the right amount of food for the household.	53.4 (1,335)	31.4 (785)	11.5 (288)	3.7 (92)	3.35	High
**Serving portions as desired and sharing excess food**					**2.79**	**Moderate**
Eat food that is about to expire or has been stored for a long time before consuming newly purchased food.	43.3 (1,083)	32.5 (811)	18.5 (463)	5.7 (143)	3.14	High
Share surplus food with those in need.	18.5 (462)	23.9 (598)	41.0 (1,025)	16.6 (415)	2.44	Moderate
Eat all the dishes that have been prepared.	60.6 (1,515)	26.8 (670)	9.7 (242)	2.9 (73)	3.45	High
Avoid leaving food on plate.	10.6 (265)	16.4 (410)	49.4 (1,235)	23.6 (590)	2.15	Moderate
**Food preservation and food reuse**					**2.14**	**Moderate**
Process surplus food through preservation methods like drying, pickling, and salting to extend its shelf life.	10.0 (250)	14.9 (373)	45.2 (1,130)	29.9 (747)	2.05	Moderate
Use surplus food to create new dishes or menus.	11.4 (285)	20.9 (523)	49.0 (1,225)	18.7 (467)	2.24	Moderate
**Other uses of food waste**					**1.91**	**Low**
Feed excess food or food scraps to animals.	25.7 (643)	21.5 (538)	31.9 (797)	20.9 (522)	2.52	Moderate
Use food scraps to produce compost.	20 (500)	14.8 (370)	26.6 (665)	38.6 (965)	2.17	Moderate
Process food scraps into bio-fermented water, which can be used as a natural fertilizer.	8.6 (215)	8.2 (205)	23.0 (575)	60.2 (1,505)	1.65	Low
Feed food scraps to earthworms to produce nutrient-rich compost.	4.4 (110)	4.3 (107)	13.4 (335)	77.9 (1,948)	1.33	Low
**Food waste conversion**					**1.17**	**Low**
Collect leftovers to produce biogas as a renewable energy source.	2.3 (57)	2.4 (60)	6.4 (160)	88.9 (2,223)	1.17	Low
**Disposal**					**1.65**	**Low**
Dispose of food waste in the household garbage can or in collection points when other options are not available.	39.1 (977)	13.7 (343)	20.1 (503)	27.1 (677)	1.65	Low
**Total**					**2.48**	**Moderate**
Cronbach alpha = .853						

Most respondents (57.1%) achieved moderate scores (26.67 to 53.33) in relation to their household food waste management practices. The rest of participants were either in the high range (40.5% scored between 53.34 and 80) or in the low range (2.40% scored between 1 and 26.66).

### Factors affecting the management of household food waste

When determining the factors influencing household food waste management, the assumptions for multiple linear regression analysis, including normality of distribution, linearity, and independence of outcome variables, were validated. The β-value (regression coefficient) was employed to determine the extent to which the independent variables possessed explanatory power. Subsequently, the relationships between the variables were analyzed. The results of this analysis, presented in Table 5, indicate that knowledge scores have a positive relationship with food waste management behavior (r = .671). Furthermore, academic qualifications, monthly income, and household size are also positively associated with food waste management behavior scores (r = .401,.508, and.210).

**Table 5:** Results of the analysis of relationships between variables.

	Gender	Academic qualifications	Monthly income	Household size	Total knowledge score	Total practice score
Academic qualifications	0.371^**^	1.000	0.047^*^	0.120^*^	0.300^*^	0.401^**^
Monthly income	0.052^**^	0.047^*^	1.000	-0.056^*^	0.309^**^	0.508^*^
Household size	0.108^**^	0.120^*^	-0.056^*^	1.000	0.127^**^	0.210^**^
Total knowledge score	0.127^**^	0.300^*^	0.309^**^	0.127^**^	1.000	0.671^**^
Total practice score	0.255^**^	0.401^**^	0.508^*^	0.210^**^	0.671^**^	1.000

* *Significance level of 0.05.*

** *Significance level of 0.01*.

In addition, a multiple regression analysis was performed to examine the relationship between the variables influencing household food waste management. The findings from this analysis are detailed in Table 6.

**Table 6:** Results of the multiple regression analysis between independent variables.

Factor		Beta	Std. Error	t	p-value
Gender		0.244	0.019	13.181	0.320
Academic qualifications		0.299^***^	0.002	15.642	0.000
Household size		0.201^***^	0.005	10.267	0.000
Monthly income		0.058^**^	0.001	2.929	0.003
Accommodation					
	Other (reference group)				
	Single house (<200 sq. m)	.028	0.021	1.358	0.175
	Single house (>200 sq. m)	.058^**^	0.017	2.859	0.004
	Townhouse	-.074^***^	0.028	-3.640	0.000
	Condominium/ apartment/ flat/ dormitory	.063^**^	0.031	-3.108	0.002
	Commercial building	-.019	0.043	-.908	0.364
Total knowledge score		.245^***^	0.006	12.649	0.000
R^2^		0.320			
Adjusted R^2^		0.300			

Note:

*  p < 0.05,

** p < 0.01,

*** p < 0.001

This study provides some insights into the factors impacting food waste management behavior. Notably, gender does not seem to exert a discernible influence on such practices. However, the knowledge variable emerges as a significant determinant, with a positive influence being observed (beta =  0.245). In addition, academic qualifications, monthly income, and household size are all factors influencing food waste management behavior (beta =  0.299, 0.058, and 0.201).

Considering the coefficient of determination (R^2^), the cumulative effect of all the independent variables in the equation explains 34.0% of the variance in the observed changes in food waste management behavior. A 68% error in the statistical model can be attributed to factors that were not captured in the variables considered in the regression.

Understanding the methods used in food waste management is crucial for mitigating improper disposal. The survey revealed that most respondents (n = 1,290, 51.6%) separated food waste into different bags before disposing of it in household bins. A similar proportion of respondents disposed of food scraps together with general waste without any separation (n = 1,210, 48.4%).

The survey also identified several obstacles that prevented households from appropriately managing food waste. In particular, 29.4% of respondents pointed to the lack of guidance from any organizations on proper food waste management practices. Additionally, 26.1% believe that such practices do not contribute to cost savings, 25.2% do not know where to share or donate excess food, and 24.2% lack knowledge about how to plan their food purchases or how to check for spoilage (Table 7).

**Table 7:** Obstacles to preventing household food waste management (more than one option) (n = 3657).

Obstacles	*n*, %
Lack of guidance from any organization on proper food waste management practices.	753 (29.4)
Perception that food prevention does not contribute to cost savings.	653 (26.1)
Uncertainty about where to share or donate excess food.	630 (25.2)
Lack of knowledge about planning food purchases and checking for spoiled food.	613 (24.2)
Lack of understanding regarding food preparation and modification techniques.	430 (17.2)
Beliefs that these practices are unnecessary due to the absence of legal mandates.	325 (13.0)
Beliefs that these practices do not significantly contribute to conserving global food resources.	253 (10.1)

The study also inquired about the obstacles encountered by households when managing food waste. A notable 43.3% of respondents were uncertain about where to go to donate or sell food scraps for the benefit of those in need or other interested parties. Many respondents (30.2%) also lacked knowledge about the methods for food waste disposal, such as composting or producing bio-fermented liquids. Moreover, a significant number of participants (23.1%) had the perception that food waste did not have value as recycled material (Table 8).

**Table 8:** Obstacles to managing household food waste (more than one option) (n = 2,991).

Obstacles	*n*, %
Uncertainty about where to donate or sell food scraps to those in need or other interested parties.	1,083 (43.3)
Lack of knowledge about the methods for household food waste disposal, such as composting or producing bio-fermented liquids.	755 (30.2)
View that food waste does not have value as recycled material.	578 (23.1)
Perception that these efforts do not lead to cost savings.	290 (11.6)
Belief that these practices are unnecessary due to the absence of legal obligations.	285 (11.4)

## Discussion

### Factors affecting household food waste management

A multivariate linear regression analysis was conducted to model self-reported practices related to household food waste management. This study revealed that educational attainment (β =  0.299), household size (β =  0.201), and monthly income (β =  0.058) are positively associated with improved household food waste practices (Table 5). These findings are consistent with demographic factors related to the reduction of food waste [22].

The study shows that higher educational levels are associated with an increase in pro-environmental behaviors within the household, a pattern that aligns with the findings of Filimonau et al. [12], Mattar et al. [13], and Abeliotis et al. [23]. This effect may be partly attributed to the fact that more educated individuals tend to have a better understanding of food labels, which enhances their ability to manage food resources effectively.

In addition, the study demonstrates that household size has a direct positive impact on improved food waste practices, with larger households wasting less food. This finding is consistent with earlier studies [11]. The rationale behind this effect is that food purchased and prepared for a large family is more likely to be shared and consumed, while smaller households would tend to waste more of it. However, it is important to note that some studies have reported contrasting results in this respect, finding that larger families waste more food than smaller ones [24,25]. Strategies to mitigate food waste in smaller households include awareness campaigns on planning and storage, small-scale technological applications, and community-sharing programs for surplus redistribution.

Moreover, the study shows a positive correlation between household income and food waste management. High-income households tend to manage food waste more effectively, as well as showing less wasteful consumption patterns. A similar effect has been previously identified by researchers in China [11] and Switzerland [26]. However, other studies indicate that higher-income households may generate more food waste [27]. This may be due to lower-income households generally producing less leftover food, while higher-income households tend to be less concerned about managing leftovers [13,28].

Studies have shown that knowledge (β =  0.245) also has a positive influence on food waste practices in households. Knowing about the various aspects of household food waste management can lead to a reduction in food waste and may help to promote proper disposal practices. This finding is aligned with the results of studies conducted in Ethiopia [29], as well as with the research by Fami et al. [24] and Visschers et al. [30]. However, some studies present differing results, indicating that knowledge may have a negative effect on food waste behavior [31]. Furthermore, other researchers suggest that awareness and knowledge of food waste are not significantly related to food waste management [32].

Interestingly, this study also found that the type of housing significantly influences food waste management behaviors. This finding is aligned with a study by Fan et al. [33] which demonstrated that certain housing types, such as single houses (over 200 square meters) (β = .058**) and condominiums/apartments/flats/dormitories (β = .063**), were positively associated with better food waste management practices. This relationship is likely due to the fact that individuals residing in single houses, who are typically homeowners, exhibit greater commitment to waste separation compared to renters [34]. In contrast, townhouses (β =  -.074***) were negatively associated with effective food waste management practices. This might be primarily due to the smaller size and space limitations of this kind of housing, which often results in a lack of designated areas for waste separation and composting. Insufficient infrastructure and a focus on general waste disposal rather than specialized food waste management might also contribute to this negative correlation. These differences underscore the importance of design considerations in enhancing waste management practices.

In general, houses with sufficient space have greater opportunities to implement waste separation, composting, and bio-extraction. Leftover food can be repurposed as pet feed, reducing pet food expenses. This practice encourages residents to actively participate in waste reduction and organic recycling [35]. Similarly, residents of condominiums and apartments, despite having limited space, often benefit from well-organized, centralized waste management systems that support recycling through building policies and coordination with local waste disposal services [36]. High-end multi-family buildings, in particular, benefit from trained staff who can effectively manage recycling [37]. In Singapore, for instance, infrastructure plays a crucial role in fostering waste separation [33].

In general, condominium owners tend to recycle more frequently than renters [38]. However, solid waste management practices in some condominium areas remain inadequate, highlighting the need for further training and regulation enforcement [39]. Policymakers should develop programs to support recycling efforts in buildings with fewer resources. Strategic implementation of clear guidelines, visual aids, and resident engagement can significantly enhance participation in recycling [36].

### Challenges and opportunities for household food waste management

This study shows that most households have insufficient food waste management habits, largely due to rapid global urbanization, which has significantly altered lifestyles. This shift has led to a decline in interest in traditional food waste disposal methods like composting, even among those with adequate space at home. The complexity, inconvenience (e.g., associated odors), and limited utility of composting in urban settings, where green spaces are scarce, further discourages its adoption. The reluctance of most modern households to practice composting underscores the broader challenges of adapting waste management strategies to urban realities.

Based on the results of this study, the primary barriers that prevent effective household food waste management include the lack of guidance from organizations (n = 753, 29.4%), the perception that such practices do not contribute to cost savings (n = 653, 26.1%), and the uncertainty about where to donate surplus food (n = 630, 25.2%). When managing household food scraps, the main challenge is that households do not know where to donate or sell them (n = 1,083, 43.3%). They also lack knowledge regarding disposal methods such as composting or bio-fermentation (n = 755, 30.2%). Moreover, the study found that most respondents only had a moderate level of knowledge regarding food waste management (n = 1,493, 59.7%). Notably, a significant proportion of individuals displayed confusion when confronted with the proposition that ‘Best before’ dates could be harmful to health (n = 1,798, 71.9%). However, this confusion was likely due to linguistic factors, as many Thai individuals do not fully grasp the nuances of English terminology, a limitation that potentially contributes to increase food waste. However, several obstacles continue to impede effective household food waste management. Challenges include the view that food waste lacks value as a recyclable material (n =  578, 23.1%), the perception that food waste management efforts do not result in significant cost savings (n =  290, 11.6%), and the view that these practices are unnecessary due to the absence of legal obligations (n =  285, 11.4%).

To address these challenges, two key actions are recommended: (1) enhancing knowledge, (2) promoting infrastructure, and (3) fostering motivation.

Firstly, numerous studies have emphasized the importance of knowledge in waste management. In particular, it is essential to promote knowledge in two key areas. Households need to be educated about the prevention of food waste and guided on how to plan their purchases and consume appropriate quantities [25,40]. They also need to understand effective food preservation techniques, including how to read the expiration labels. In addition, it is important to enhance households’ knowledge about food waste management, especially where homes have sufficient space or in rural areas, by introducing simple composting techniques and providing accessible composting kits and adequate guidance [41,42]. These initiatives will enhance household awareness of food waste prevention and management, contributing to improved efficiency in the food supply chain while minimizing surplus food production and waste [25,41,42].

Educational efforts should target all age groups, with a special focus on teenagers [43]. Malaysia, a nation with a socio-economic context comparable to Thailand, has recently implemented a school-based pilot project that has improved community-level food waste management. This project, which promoted the transformation of food waste into organic fertilizer, generated significant income and showed that it could be potentially scalable at the national level. The success of the project underscores the importance of targeting adolescents in educational initiatives, given their pivotal role in driving behavioral change [44]. These initiatives can be implemented through social media, school curricula, or local training programs [45].

Secondly, promoting infrastructure aligned with the type of housing, particularly through public-private-people partnerships, is another key area of improvement. According to the Department of Pollution Control, Thailand faces significant challenges in waste management infrastructure. At the moment, the country only has three composting or biogas systems and 114 scientifically approved waste disposal sites (5.48%). The remaining 1,965 sites (94.52%) operate as open dumps or controlled landfills [17]. A similar situation is observed in India, as shown by [46]. Consequently, government agencies should collaborate with the private sector to provide adequate facilities, especially in condominiums, where waste disposal zones must be carefully designed [34,47–49]. Improper food waste management can lead to widespread odors and numerous hygienic issues. While high-end buildings may already support recycling, policymakers need to create programs to assist those with fewer resources [37] and promote composting solutions tailored to urban contexts, such as community composting centers or indoor odor-controlled composting systems like onsite aerobic food waste (FW) digestion [50]. This approach, by encouraging greater household waste segregation and recycling, can support effective waste management plans based on the concept of a circular economy.

Thirdly, fostering public participation requires the promotion of initiatives that integrate cultural, religious, and economic incentives appropriate to the Thai context as well as actions that address inconsistent government policies. These efforts should begin by aligning the Food Waste Action Plan Phase 1 (2023–2027) with the broader Waste Action Plan Phase 2 (2023–2027) to develop a unified approach to food waste management, which includes the enforcement of existing laws and imposes penalties for non-compliance. Even when food waste is perceived as having no tradable or recyclable value [51], economic incentives such as rewards for food waste reduction or subsidies for composting equipment [52] can encourage households to adopt sustainable practices.

Additionally, leveraging Thai cultural traditions in rural areas, for example by using surplus food to feed animals [53] or embracing Buddhist principles like merit-making through donations and surplus food sharing [54] can further motivate participation. In particular, religious beliefs play a crucial role in fostering environmental awareness, which in turn contributes to reducing food waste [55]. By integrating these diverse motivating factors, this approach can advance sustainable household food waste management practices that align with Thailand’s cultural traditions and policy frameworks.

The main limitation of this study is that it has excluded psychological variables from the questionnaire. As the study was conducted nationwide, the inclusion of too many questions could have discouraged respondents from fully participating in the survey. Moreover, the reliance on a quantitative approach inherently limits the ability to explore the nuanced reasons underpinning behavioral differences across diverse demographic and social groups. Future research should adopt a mixed-methods approach, incorporating in-depth interviews or focus groups, as a way to provide richer insights into the underlying motivations and barriers to effective food waste management practices among diverse populations. The combination of methods would offer deeper insights into the motivations and barriers influencing food waste management practices. Furthermore, future studies should investigate the whole range of cultural, psychological, and attitudinal factors in order to provide a more comprehensive understanding of household waste management behaviors. Developing a more comprehensive understanding of these aspects will help policymakers in designing targeted interventions that address both structural and behavioral dimensions of food waste reduction.

## Research implications and conclusions

### Theoretical implications

Theoretically, these findings contribute to a growing body of literature that emphasizes the significance of socio-economic factors in household food waste management especially education, household size, and income. It emphasizes the need to increase environmental knowledge and food waste management at the household level. The positive relationship between large households and reduced waste supports socio-economic theories about food sharing. In addition, the link between large residential areas and improved food waste practices highlights the importance of this connection to the broader theoretical debate on the intersection of environmental behavior and urban design.

### Policy implications

Thailand has never had a specific food waste management policy, relying instead on its National Solid Waste Management Master Plan (2016–2021). The findings of this study, which analyzed data from 2,500 respondents nationwide, have contributed to the first road map on food management in Thailand—the Roadmap on Food Waste Management (2023–2030) and the Action Plan on Food Waste Management Phase 1 (2023–2027) [17].

The findings of the study highlight the need for tailored interventions in urban and rural areas. Rural strategies should focus on access to composting technologies and biogas systems, while leveraging cultural and religious values to promote sustainability. Urban initiatives require compact, odor-controlled composters or community composting centers, supported by incentives like tax rebates and reduced waste collection fees.

This study bridges the gap between socio-demographic and knowledge-based variables and practical interventions. As the study has shown, households with higher income and educational attainment exhibit superior waste management practices, indicating that targeted educational campaigns can address gaps among lower-income groups. The impact of housing also underscores the need for tailored solutions, such as promoting composting technologies in rural households with ample space, centralized waste sorting systems, and odor-controlled composters in urban areas. Moreover, increased knowledge of food waste management—a significant predictor of improved practices—can be bolstered through school programs, local workshops, and social media campaigns. These efforts can directly link the enhancement of knowledge to the improvement of behavior and public awareness. By translating these findings into specific, evidence-based strategies, the study equips policymakers and community leaders with practical tools to address both structural and behavioral barriers, fostering sustainable waste management practices at the community level.

The enhancement of appropriate knowledge and infrastructure improvement can be achieved through public-private partnerships. This is essential for the efficient disposal of waste. The insights gained from this research at a national level will provide the foundation for the development of a comprehensive food waste management framework in Thailand and provide valuable information for other developing countries facing similar urban growth challenges.

## Supporting information

S1 File Original survey questionnaire used in the study.
(DOCX)
