# Supplementary material for: Socio-demographic drivers of household food waste management practices in Thailand
Source: PLoS One. 2025 Apr 1;20(4):e0321054. doi: 10.1371/journal.pone.0321054 (PMC11960981; doi:10.1371/journal.pone.0321054)
Supplement: S1 File — (DOCX) [file pone.0321054.s001.docx]

**S1 File. Original survey questionnaire used in the study**

**Section 1** Demographic characteristics

Gender ❒ Male ❒ Female ❒ Other

Academic qualifications ❒ No education ❒ Primary school ❒ Lower secondary education ❒ Upper secondary education ❒ diploma or vocational certificate ❒ Bachelor's degrees ❒ Postgraduate degree

Martial status ❒ Single ❒ Married ❒ Widowed or divorced

Household size ❒ 1 ❒ 2-5 ❒ 6-9 ❒ 10 or more

Monthly income ❒ Less than 15,000 baht (< $424) ❒ 15,001 – 30,000 baht ($424-$848) ❒ 30,001 – 45,000 baht ($849-$1,272) ❒ 45,001 – 60,000 baht ($1,273-$1,697) ❒ 60,001 – 75,000 baht ($1,698-$2,121) ❒ More than 75,000 baht (> $2,121)

**Accommodation** ❒ Single house (< 200 sq. m) ❒ Single house (> 200 sq. m) ❒ Condominium/ Apartment/ Dormitory/Flat ❒ Townhouse ❒ Commercial building ❒ other………………

**Section 2** Knowledge of household food waste management

| **Knowledge question** | **True (*n*, %)** | **False (*n*, %)** |
| --- | --- | --- |
| Food waste consists of food scraps that are no longer edible. |  |  |
| Food that is still edible but is thrown away is not considered food waste. |  |  |
| Consuming food past the ‘Best before’ date can be harmful to health. |  |  |
| Food preservation and processing can reduce the amount of food waste generated in households. |  |  |
| Food waste has no impact on waste storage because it can be easily decomposed. |  |  |
| Wastewater from decomposing food discarded by households causes soil and water pollution. |  |  |
| Planning purchases and avoiding hoarding can help reduce food waste in households. |  |  |
| Sharing food with those in need can decrease household food waste. |  |  |
| Composting discarded food can further reduce household food waste. |  |  |
| Using discarded food to raise animals can reduce the amount of food waste in households. |  |  |

**Section 3** Summary of household food waste management practices by frequency

| **Practices** | **Always** | **Often** | **Sometimes** | **Never** |
| --- | --- | --- | --- | --- |
| **Planned purchasing** |  |  |  |  |
| Regularly check the refrigerator and dry food locker before dispensing. |  |  |  |  |
| Write a food list of necessary food purchases before going to the market. |  |  |  |  |
| **Purchasing food as needed** | | | | |
| Buy food according to the planned purchase list. |  |  |  |  |
| Purchase the right amount of food to avoid hoarding large quantities. |  |  |  |  |
| **Proper food storage** |  |  |  |  |
| Always store newly purchased food inside the refrigerator and move pre-stored food outside. |  |  |  |  |
| Regularly check the expiration dates and prioritize using food before it expires. |  |  |  |  |
| **Preparing appropriate quantities of food** | | | | |
| Cook with existing ingredients before buying new ones. |  |  |  |  |
| Prepare the right amount of food for the household. |  |  |  |  |
| **Serving portions as desired and sharing excess food** | | | | |
| Eat food that is about to expire or has been stored for a long time before consuming newly purchased food. |  |  |  |  |
| Share surplus food with those in need. |  |  |  |  |
| Eat all the dishes that have been prepared. |  |  |  |  |
| Avoid leaving food on plate. |  |  |  |  |
| **Food preservation and food reuse** | | | | |
| Process surplus food through preservation methods like drying, pickling, and salting to extend its shelf life. |  |  |  |  |
| Use surplus food to create new dishes or menus. |  |  |  |  |
| **Other uses of food waste** | | | | |
| Feed excess food or food scraps to animals. |  |  |  |  |
| Use food scraps to produce compost. |  |  |  |  |
| Process food scraps into bio-fermented water, which can be used as a natural fertilizer. |  |  |  |  |
| Feed food scraps to earthworms to produce nutrient-rich compost. |  |  |  |  |
| **Food waste conversion** | | | | |
| Collect leftovers to produce biogas as a renewable energy source. |  |  |  |  |
| **Disposal** | | | | |
| Dispose of food waste in the household garbage can or in collection points when other options are not available. |  |  |  |  |

**Section 4** **Obstacles to preventing household food waste management (more than one option)**

| **Obstacles** | ***n*, %** |
| --- | --- |
| Lack of guidance from any organization on proper food waste management practices. |  |
| Perception that food prevention does not contribute to cost savings. |  |
| Uncertainty about where to share or donate excess food. |  |
| Lack of knowledge about planning food purchases and checking for spoiled food. |  |
| Lack of understanding regarding food preparation and modification techniques. |  |
| Beliefs that these practices are unnecessary due to the absence of legal mandates. |  |
| Beliefs that these practices do not significantly contribute to conserving global food resources. |  |

**Section 5** **Obstacles to managing household food waste (more than one option)**

| **Obstacles** | ***n*, %** |
| --- | --- |
| Uncertainty about where to donate or sell food scraps to those in need or other interested parties. |  |
| Lack of knowledge about the methods for household food waste disposal, such as composting or producing bio-fermented liquids. |  |
| View that food waste does not have value as recycled material. |  |
| Perception that these efforts do not lead to cost savings. |  |
| Belief that these practices are unnecessary due to the absence of legal obligations. |  |
